# Effects of ulinastatin and docataxel on breast tumor growth and expression of IL-6, IL-8, and TNF-α

**DOI:** 10.1186/1756-9966-30-22

**Published:** 2011-02-23

**Authors:** Xiaoliang Zhao, Xin Sun, Feng Gao, Jie Luo, Zhijun Sun

**Affiliations:** 1 Department of Breast, Pancreas, and Thyroid Surgery; Second Affiliated Hospital of Chongqing Medical University, 74 Lingjiang Road, Yuzhong District, Chongqing 400010, PR China

## Abstract

**Objective:**

This study investigated the effects of Ulinastatin (UTI) and docataxel (Taxotere, TAX) on tumor growth and expression of interleukin-6 (IL-6), interleukin-8 (IL-8), and tumor necrosis factor-α (TNF-α) in breast cancer.

**Methods:**

MDA-MB-231 human breast carcinoma cells were cultured in vitro and injected into nude mice to establish breast tumor xenografts in vivo. Cultured cells and mice with tumors were randomly divided into four groups for treatment with TAX, UTI, and TAX+UTI. The effects of these drug treatments on cell proliferation and apoptosis was measured using the MTT assay and the Annexin V/propidium iodide (PI) double-staining method, respectively. IL-6, IL-8, and TNF-α expression levels were determined by measuring mRNA transcripts in cultured cells by RT-PCR and cytokine proteins in solid tumors using immunohistochemistry.

**Results:**

UTI, TAX, and UTI+TAX inhibited the growth of MDA-MB-231 cells in vitro and tumors in vivo. These two drugs, particularly when used in combination, promote tumor cell apoptosis and down-regulate the expression IL-6, IL-8, and TNF-α cytokines.

**Conclusion:**

Both UTI and TAX inhibited the growth of MDA-MB-231 breast carcinoma cells. UTI enhanced the inhibitory effect of TAX by a mechanism consistent with the down-regulated expression of IL-6, IL-8, and TNF-α.

## Backgroud

Along with the increasing incidence of breast cancer tumors, which now account for 18% of all female tumors, 1.2 million women suffer from breast cancer worldwide. Many important problems pertaining to the oncological details of invasion and metastasis pose significant challenges to scientists.

With the development of new techniques in molecular biology, further exploration into the mechanisms related to the occurrence of breast cancer have become a hotspot in the field of cancer research. The cytokines, which play regulatory roles in disease development have become an important topic for many researchers. IL-6, IL-8, and TNF-α are one group of cytokines produced by mononuclear macrophages and endotheliocytes involved in activating and inducing T cells, B cells, and natural killer cells to target and phagocytosize pathogenic cells. Additionally, these cytokines are important factors in inflammation and pathophysiology.

In this study, we monitored the effects of UTI and TAX, individually and in combination, on the growth of the negative estrogen receptor (ER-) human breast carcinoma cell line, MDA-MB-231. Using both cultured cells *in vitro *and xenografted tumors *in vivo*, we also examined the effects of UTI and TAX on apoptosis and the expression levels of IL-6, IL-8, and TNF-α cytokines.

## Materials and methods

### 1.1 Cell lines and animals

The human breast cancer cell line MDA-MB-231(ER-) was a generous gift from the Shanghai Institutes for Biological Sciences, Chinese Academy of Sciences (CAS). Fifty female BALB/c-nu/nu nude mice, 5 weeks old and weighing 17-21 g, were purchased from the Beijing Institute of Experimental Zoology, CAS, and maintained in the Chongqing Medical University Animal Research Center (production license No. SCXK (Jing), 2005-0014, usage permit No. (Yu), 2007-0001).

### 1.2 Reagents

UTI was kindly provided by Techpool Bio-Pharma Co., Ltd. TAX was a generous gift from Sanofi-aventis Pharma Co., Ltd. Maxima™ SYBR Green/ROX qPCR Master Mix (2X) and RevertAid™ First Strand cDNA Synthesis Kits was purchased from Fermentas Co. Ltd., Canada; Trizol kit was purchased from Invitrogen Co, Ltd; RT-PCR kit was purchased from NanJing KeyGen Biotech Co, Ltd. MTT ((3-(4,5-dimethylthiazol-2-yl)-2,5-diphenyltetrazolium bromide), dimethyl sulfoxide (DMSO), propidium iodide(PI), and phosphate buffered saline (PBS) were purchased from Sigma Chemical Co., Ltd. AMV reverse transcriptase was purchased from Promega Co, Ltd; RPMI-1640 was purchased from GIBCO Co., USA. The secondary antibody kit and diaminobenzidine (DAB) chromogenic substrate were purchased from Zhongshan Goldenbridge Biotechnology Co., Ltd. Vascular endothelial growth factor-C (VEGF-C), basic fibroblast growth factor (bFGF), and nerve growth factor (NGF) primary antibodies were purchased from Abcam Co., Ltd., UK.

### 1.3 Cell cultures and nude mice

MDA-MB-231 cells were cultured in RPMI-1640 medium containing 10% fetal bovine serum (FBS), 100 U/mL of penicillin, and 100 U/mL of streptomycin at 37°C in a 5% CO_2 _atmosphere. Following propagation for 2-3 days, cells in logarithmic growth phase were digested with 1.0 mL of 0.25% trypsin for 2-3 min, separated from trypsin, and incubated with double antibody solution in RPMI-1640 medium containing 10% FBS. Nude mice were housed in a specific pathogen free (SPF) environment at 22-25°C and 50-65% relative humidity with sterile drinking water, food, and experimental equipment.

### 1.4 Experimental groups and drug treatments

Cultured MDA-MB-231 cells were divided into four random groups: Control (RPMI-1640 medium alone), UTI (8000 U/mL), TAX (3.7 ug/mL; 5 × 10^-6 ^M), and UTI+TAX. MDA-MB-231 cells were harvested, rinsed twice in PBS, resuspended in serum-free RPMI-1640 medium at a density of 2.5 × 10^10 ^cells/L, and inoculated into the right axillary breast tissue of nude mice (0.2 mL/mouse × 50 mice). At 21 days post-inoculation, 29 mice with tumors ≥ 500 mm^3 ^were divided into four experimental groups: 1) Control (8 mice injected with PBS); 2) UTI (7 mice injected with 8000 U/mL UTI); 3) TAX (7 mice injected with 20 mg/kg TAX); and 4) UTI+TAX (7 mice injected with both UTI and TAX as in groups 2 and 3). All inoculations were i.p. For groups 1 and 2, 0.2 mL was injected per mouse every day for 20 days. For groups 3 and 4, 20 mg/kg was injected on days 1, 7, and 14. After 21 days, the mice were sacrificed for sample preparation. The maximum length (L) and the minimum diameter (D) of each tumor was measured using vernier calipers to calculate the tumor volume (cm^3^). Tumor growth curves were constructed and tumor growth rates were calculated for each experimental group. We validated the synergistic or antagonistic effects of the drugs by calculating the q value using King's formula. Synergistic, additive, or antagonistic effects were determined by q > 1.15, 1.15 > q > 0.85, q < 0.85, respectively. The formulas used were: tumor volume (cm^3^) = (L2 × D)/2; tumor growth inhibition rate(%) = [1-(V1-V2)/(V3-V4)] × 100%, where V1 and V2 are the respective starting and ending average tumor volumes in the drug-treated groups and V3 and V4 are the respective starting and ending tumor volumes in the control group; and q = Ea+b/[(Ea+Eb)-Ea × Eb], where Ea, Eb, (Ea+Eb) represent the inhibitory rates of UTI, TAX, and UTI+TAX, respectively (King's formula).

### 1.5 Quantitation of cell proliferation using the MTT assay

Cells were seeded into 96-well plates at a density of 4 × 10^3 ^cells per 200 μL per well. The cells were divided into four experimental groups (6 wells/group) as described in 1.4.1 and cultured in RPMI-1640 + 10% FBS. After 24, 48, and 72 h, 20 μL of 5 mg/mL MTT was added to each well for 4 h. Then 150 μL of DMSO was added to each well with shaking for 10 min. The absorbance (A) at 570 nm was measured using an enzyme-linked immunosorbant assay (ELISA) plate reader to quantitate the inhibitory rate. The experiment was repeated three times. Inhibitory rate (%) = (1-experimental group A_570_/control group A_570_) × 100%

### 1.6 MDA-MB-231 cell apoptosis

Adherent MDA-MB-231 cells were detached from their substrates by digestion with 0.125% EDTA-free typsin, centrifuged for 5 min, resuspended, and rinsed by centrifugation in PBS at 4°C. The cell pellet was resuspended in 490 μL PBS containing 5 μL of FITC-Annexin and 5 μL of 250 ug/mL PI and incubated on ice for 10 min. After two rinses, the cells were analyzed by flow cytometry using a FACS Vantage SE from Becton-Dickinson, USA.

### 1.7 Detection of IL-6, IL-8, and TNF-α mRNA transcripts by RT-PCR

Based on the complete nucleotide sequences of IL-6, IL-8, TNF-α, and control gene β-actin supplied by GenBank, Primer 5.0 software was used by Nanjing Keygen Biotech Co. Ltd. to design and synthesize primers for reverse transcriptase-polymerase chain reaction (RT-PCR). The product lengths for IL-6, IL-8, TNF-α, and β-actin were 84, 160, 108, and 136 base pairs, respectively. The primer pairs used were:

IL-6 sense: 5' AAATTCGGTACATCCTCGAC 3',

IL-6 anti-sense: 5' CCTCTTTGCTGCTTTCACAC 3',

IL-8 sense: 5' TACTCCAAACCTTTCCACCC 3', IL-8 anti-sense: 5' AAAACTTCTCCACAACCCTC 3',

TNF-α sense: 5' GCCTGCTGCACTTTGGAGTG 3', TNF-α anti-sense: 5' TCGGGGTTCGAGAAGATGAT 3', β-actin sense: 5' GCAGAAGGAGATCACAGCCCT 3', and β-actin anti-sense:5' GCTGATCCACATCTGCTGGAA 3'.

The SYBR Green/ROX qPCR master mix was used with initial denaturation at 95°C for 5 min followed by: 45 cycles of denaturation at 94°C for 15 s; annealing at 60°C for 30 s; and extension at 55°C for 1 min, and 1 min extension at 95°C. The luminescence signal was measured during the extension process. The transcritical cycle (Ct) was analyzed using the PCR apparatus procedure and copy numbers were calculated from 2^-ΔΔCt^, the copy number ratio of expanding target genes and the internal control gene (β-actin) to determine the mRNA expression levels of the target genes.

### 1.8 Detection of IL-6, IL-8, and TNF-α cytokines in xenografted tumors by immunohistochemistry

Carcinoma tissues were dehydrated using a graded series from 75, through 80 and 95, to 100% ethanol. Dehydrated samples were completely immersed in wax, cut into 5 μm sections, and mounted on 3-triethoxysilylpropylamine (APES)-treated glass. Sections were treated with 50 μL non-immune animal serum plus 50 μL of a 1:50 dilution of anti-IL-6, IL-8, and TNF-α antibodies for 10 min. PBS was used as a negative control. Primary antibody incubations were followed by 50 μL of biotin-labeled secondary antibody and 50 μL of streptavidin-peroxidase (SP) solution for 10 min. The sections were rinsed with PBS three times for 3 min and 100 μL of fresh DAB chromogenic substrate solution was added. Sections were examined microscopically for color development for 5-10 min, redyed with hematoxylin (HE), re-blued with saturated lithium carbonate, dehydrated with the graded ethanol series (as above), and sealed in neutral gum.

Imaging of all immunohistochemical sections was performed using a Leica microscope electronic imager. The appearance of tan color or tan particles indicated a positive reaction in the cells. We performed IOD analysis on the sections in each group using Image Pro-plus v6.0 software to compare the differences between the group.

### 1.9 Statistical analysis

All data were analyzed using PASW 18.0 software and represented as $\bar{x}\pm \text{s}$. The variance analysis was adopted for comparisons between groups. P < 0.05 was considered to be statistically significant.

## Results

### 2.1 Effects of UTI and TAX on MDA-MB-231 cell proliferation

Relative to the control group, the growth of MDA-MB-231 cells treated with UTI, TAX, and UTI+TAX for 24 h was significantly inhibited (P < 0.05; Table 1). The inhibitory effect increased in a time-dependent manner when the cells were treated for 48 and 72 h (P < 0.01; Table 1). The strongest inhibitory effect was produced by co-treatment with both drugs and the weakest effect occurred with UTI alone (UTI+TAX > TAX > UTI). The differences were statistically significant (P < 0.01; Table 1).

**Table 1:** Effects of UTI and TAX on the proliferation of human breast cancer MDA-MB-231 cells *in vitro *(A_570_, $\bar{x}\pm s$)

	24 h		48 h		72 h	
	
Groups	A value ($\bar{x}\pm s$)	Inhibition rate (%)	A value ($\bar{x}\pm s$)	Inhibition rate (%)	A value ($\bar{x}\pm s$)	Inhibition rate (%)
Control	1.086 ± 0.082	0	1.366 ± 0.042	0	1.881 ± 0.106	0
UTI	1.000 ± 0.067^a^	7.919	0.867 ± 0.102^a^	36.530	0.631 ± 0.067^a^	66.454
TAX	0.853 ± 0.051^a,b^	21.455	0.703 ± 0.043^a,b^	48.536	0.440 ± 0.063^a,b^	76.608
UTI+TAX	0.773 ± 0.041^a,b,c^	28.821	0.590 ± 0.059^a,b,c^	56.808	0.315 ± 0.068^a,b,c^	83.254

^a ^*P *< 0.05 for all treatment groups versus control; ^b ^*P *< 0.01 for TXT and UTI+TAX groups versus UTI group; ^c ^*P *< 0.01 for UTI+TAX group versus TAX group.

### 2.2 Effects of UTI and TAX on MDA-MB-231 cell apoptosis

Compared to the control group (1.00), the level of apoptosis increased to 1.84 for the UTI group, 3.90 for the TAX group, and 6.79 for the UTI+TAX group (Table 2).

**Table 2:** Apoptosis of MDA-MB-231 cells treated with different drugs

Treatment	Apoptotic rate(%)	Fold increase
Control	2.52 ± 0.53	0
UTI	7.16 ± 1.59	1.84
TAX	12.35 ± 1.88	3.90
UTI+TAX	19.64 ± 2.26	6.79

Data expressed as mean ± sd. Note: p < 0.05 among different treatments.

### 2.3 Expression of IL-6, IL-8, and TNF-α mRNA in MDA-MB-231

**Figure 1:**
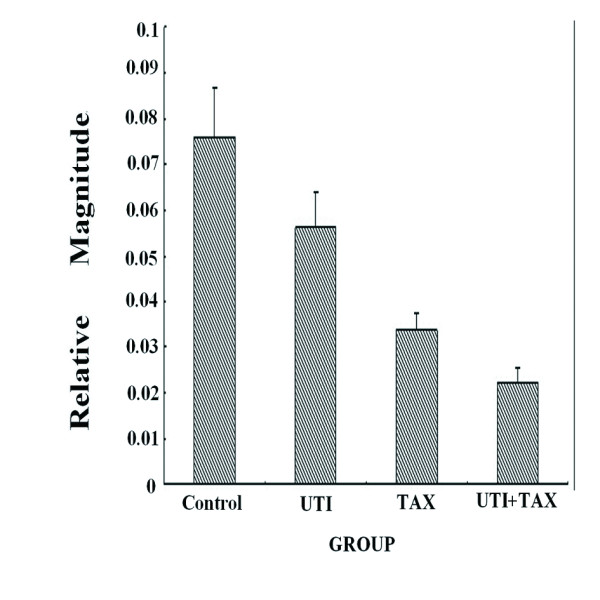
**Effects of UTI and TAX on IL-6 mRNA levels in MDA-MB-231 cells**.

**Figure 2:**
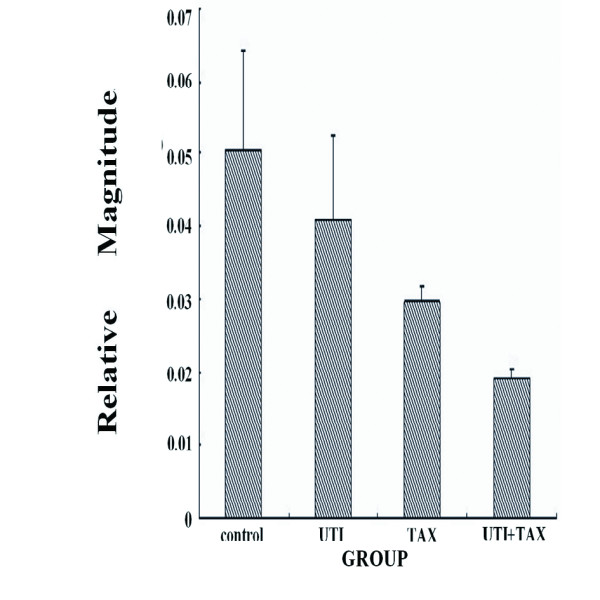
**Effects of UTI and TAX on IL-8 mRNA levels in MDA-MB-231 cells**.

**Figure 3:**
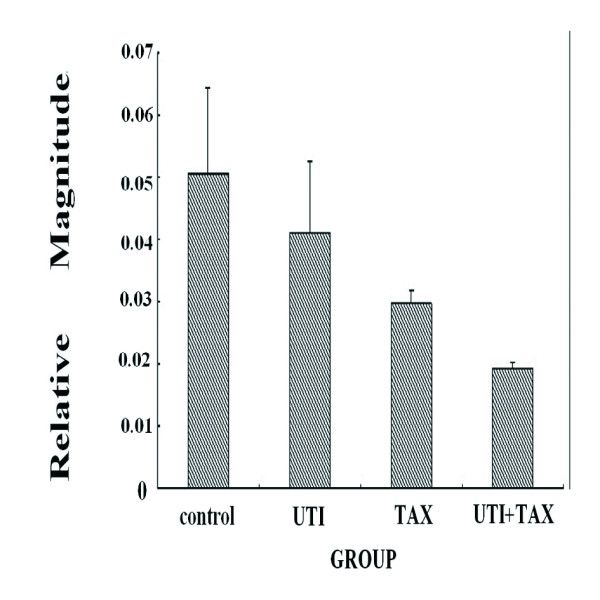
Effects of UTI and TAX on TNF-α mRNA levels in MDA-MB-231 cells.

Treatment of MDA-MB-231 cells with both UTI and TAX down-regulated the expression of IL-6, IL-8, and TNF-α transcripts greater than treatment with either UTI or TAX alone (P < 0.05; Figure 1, Figure 2, Figure 3).

### 2.4 Effects of UTI and TAX on the growth of ed breast tumor xenografts

**Figure 4:**
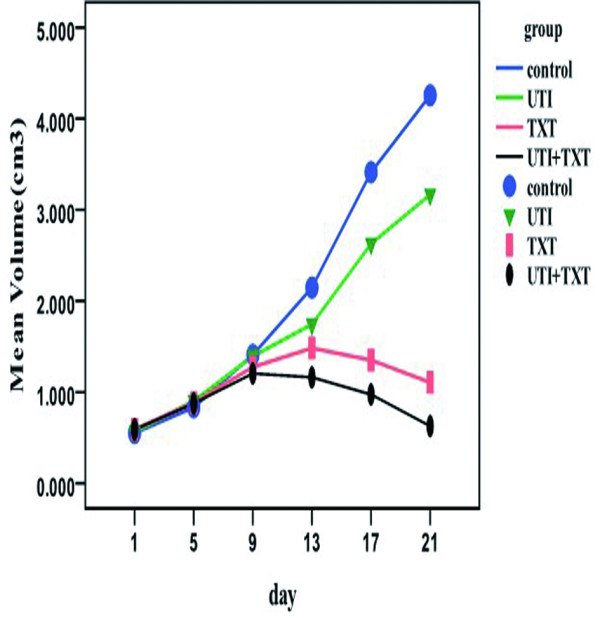
Effects of UTI and TAX on transplanted breast tumor size in nude mice

One mouse in the control group died on day 13 and one mouse in the UTI group died on day 18 due to consumption and cachexia. The 7 tumors in the control group enlarged in a time-dependent manner, with no spontaneous tumor deflation or regression. For the 6 mice in the UTI group, the volume of their xenografted tumors gradually increased at a rate less than that of the mice in the control group (P < 0.05). For the 7 mice in the TAX group, the volume of their xenografted tumors also gradually decreased relative to the controls. For the 7 mice in the UTI+TAX group, the volume of their tumors decreased with the greatest rate and extent over time (P < 0.05; Table 3; Figure 4).

**Table 3:** Effects of UTI and TAX on the weight and restraining rate of breast tumor xenografts in nude mice

Group	Sample size(n)	Mean tumour volume before treatment(cm^3^)	Mean tumour volume after treatment(cm^3^)	Mean tumour inhibition(%)
Control	7	0.551 ± 0.026	4.257 ± 0.212	0
UTI	6	0.563 ± 0.012	3.166 ± 0.134	29.312
TAX	7	0.592 ± 0.018	1.106 ± 0.145	86.021
UTI+TAX	7	0.589 ± 0.021	0.627 ± 0.016	98.264

### 2.5 Effects of UTI and TAX on the expression of IL-6, IL-8, and TNF-α proteins in breast tumor xenografts

**Figure 5:**
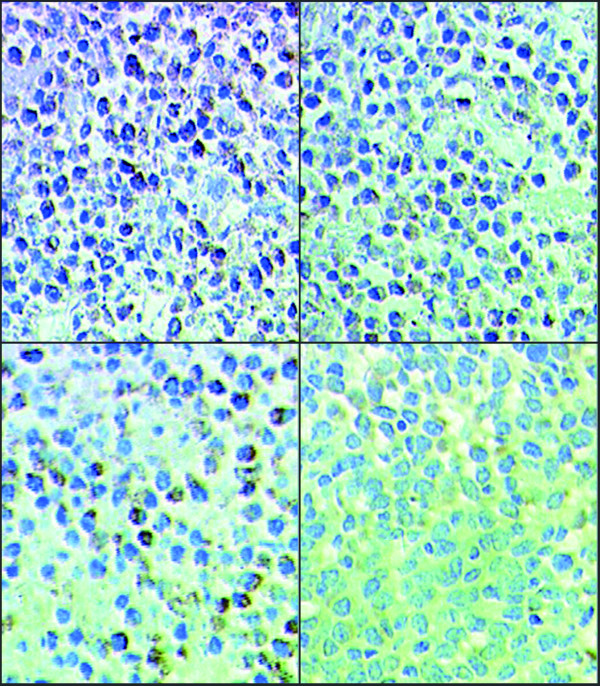
**Effects of UTI and TAX on IL-6 protein expression in human breast cancer xenografts in immunohistochemistry**: 1. Control group SP × 400 2. UTI group SP × 400, 3 TAX group SP × 400 4. UTI+TAX group SP × 400

**Figure 6:**
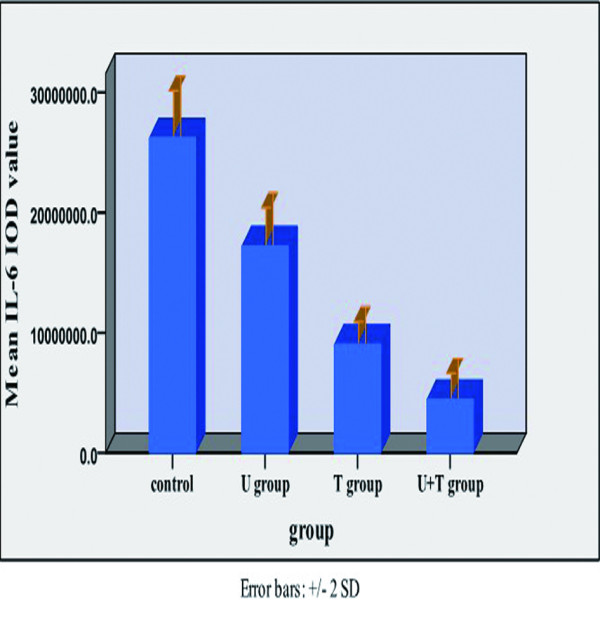
Effects of UTI and TAX on IL-6 protein expression in human breast cancer xenografts in histogram

**Figure 7:**
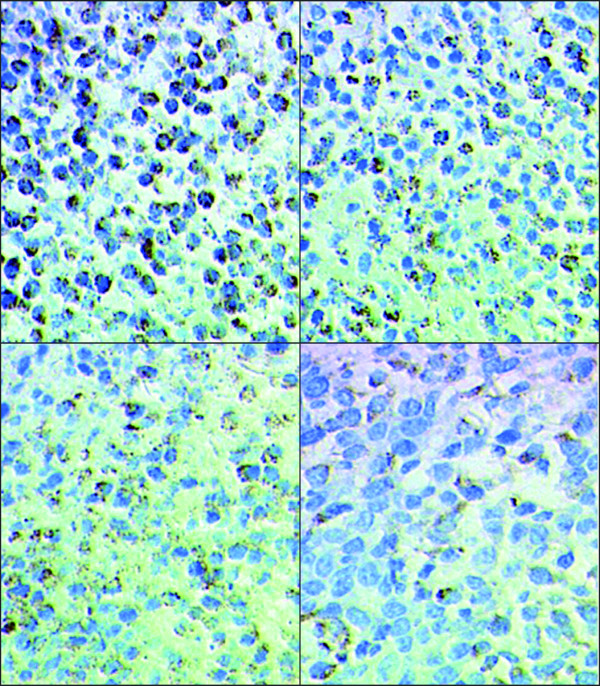
**Effects of UTI and TAX on IL-8 protein expression in human breast cancer xenografts in immunohistochemistry**: 1. Control group SP × 400 2. UTI group SP × 400, 3 TAX group SP × 400 4. UTI+TAX group SP × 400

**Figure 8:**
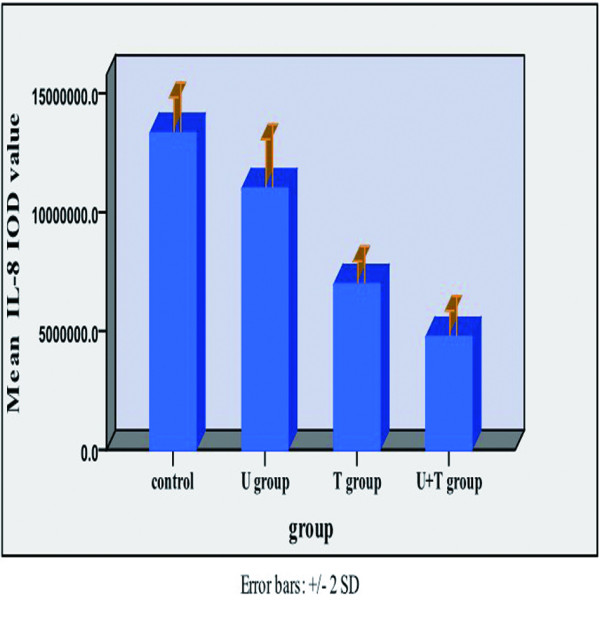
Effects of UTI and TAX on IL-8 protein expression in human breast cancer xenografts in histogram

**Figure 9:**
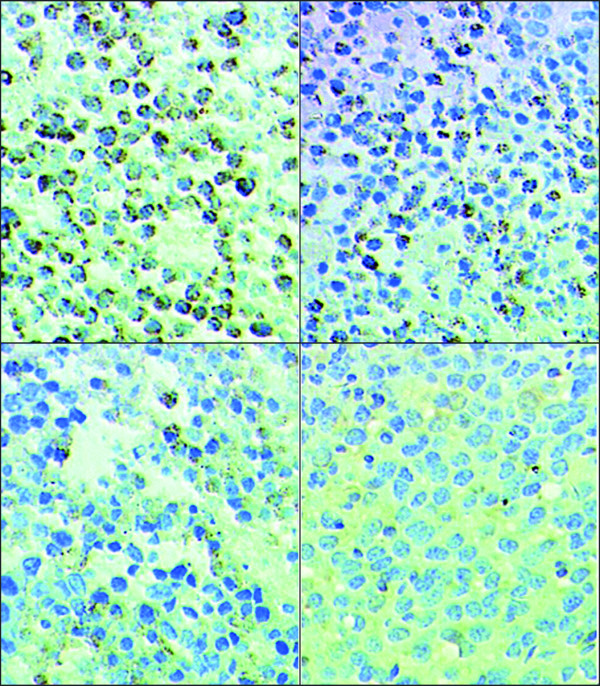
**Effects of UTI and TAX on of TNF-α protein expression in human breast cancer xenografts in immunohistochemistry**: 1. Control group SP × 400 2. UTI group SP × 400, 3 TAX group SP × 400 4. UTI+TAX group SP × 400

**Figure 10:**
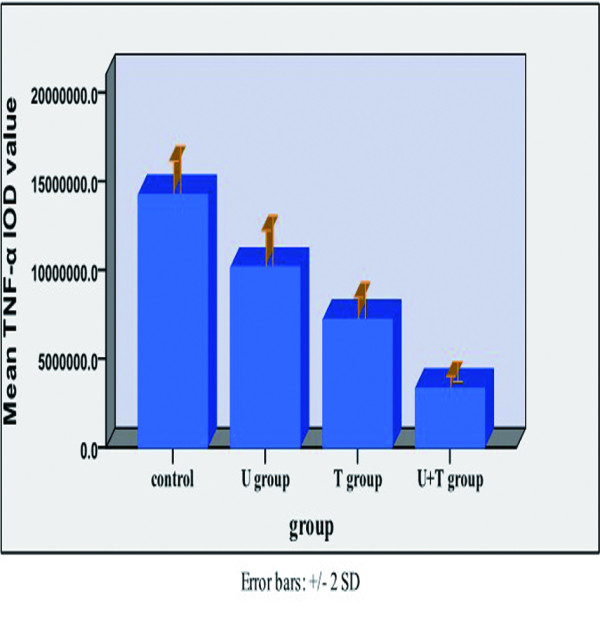
Effects of UTI and TAX on of TNF-α protein expression in human breast cancer xenografts in histogram

Relative to untreated MDA-MB-231 tumor xenografts, the xenografts from mice treated with UTI, TAX, and UTI+TAX showed decreased expression of IL-6 (Figure 5, Figure 6), IL-8 (Figure 7, Figure 8), and TNF-α (Figure 9 Figure 10) proteins. Treatment with UTI+TAX decreased cytokine expression greater than treatment with either UTI or TAX alone (P < 0.01; Figures. 5,6,7,8,9,10).

## Discussion

Ulinastatin (UTI) is a serine protease inhibitor (SPI) with extensive inhibitory effects on cell proliferation and extracellular matrix degradation. Consequently, the protection of patients in radiotherapy and chemotherapy becomes an important consideration for researchers. The experiment of Kobayashi [1] showed that UTI inhibited human ovarian cancer and the effect could be related to UTI down-regulation of protein kinase C (PKC), which regulates the methionine/extracellular-signal of the MEK/ERK/c-Jun-dependent signal pathway to collaboratively down-regulate the plasminogen activator urokinase. The application of UTI and etoposide can enhance the inhibition of metastasis in Lewis lung carcinoma (3LL) [2]. Our experiments show that UTI can inhibit the growth of xenografted breast carcinoma tumors with the co-application of both UTI and TAX being most effective.

As one of the core cytokines, interleukin-6 (IL-6), is produced by lymphocytes, mononuclear cells, fibroblasts, vascular endothelial cells, and some cancer cells, primarily in autocrine and paracrine secretions. After secretion, IL-6 combines with the α-subunit of the membrane-bound IL-6 receptor (IL-6R) and the β-subunit of glycoprotein 130 (gp 130) for cell signaling. Goswami [3] used an anti-IL-6 primary antibody to inhibit the proliferation of human glioblastoma multiforme cells, demonstrating that IL-6 has some effect on promoting tumor cell proliferation. Burger [4] also reported that cancer cells and tumor-related macrophages can release high concentrations of IL-6. Hussein [5] showed that high-levels of IL-6 indicate poor prognosis and the concentration of IL-6 in the serum of breast cancer patients is not only elevated, but increases with the clinical stage of breast cancer. Sasser [6] found that the growth rate of MCF-7 estrogen-receptor-positive (ER+) breast carcinoma cells doubled *in vitro *and increased even more *in vivo *following treatment with recombinant human IL-6. Our results show that UTI inhibits the expression of IL-6.

Interleukin-8 (IL-8) is produced by monocytes, macrophages, T cells, and vascular endothelial cells. UTI enables neutrophil chemotaxis, defluvium, and lyase release. Additionally, UTI can protect against inflammation, promote T cell chemotaxis, and reinforce the immune response. Heideman [7] suggested that IL-8 promotes leukin chemotaxis into tumors, leading to tumor neovascularization and the acceleration of tumor growth and metastasis. IL-8 enters cells by combining with the chemokine receptor CXCR1, to activate the extracellular ERK2/1 signaling pathway and promote the formation of new microvessels. It has been reported that the expression of IL-8 in breast carcinoma cells is inversely proportional to the level of estrogen receptors (ER). Based on this relationship, decreased expression of ER increases the expression of IL-8, leading to increased tumor deterioration [8]. Our prophase experiment showed that UTI can inhibit the expression of CXCR4 [9], which is produced by stroma derived factor-1. In the present study, UTI and TAX inhibited the expression of IL-8 in xenografted breast tumors in nude mice.

TNF-α is a peptide hormone that affects tumor cell necrosis, inflammation, and the immune response. The effects of TNF-α are widespread and mediated through nearly all of the TNF-α receptors on tumor cells and many other cells. Gong [10] demonstrated that increased TNF-α promotes invasion and metastasis in ductal carcinomas in a scalar fashion. The TNF secreted by tumor-related macrophages can enhance the invasion of tumors by increasing the expression of matrix metalloproteases (MMPs) in breast carcinoma and vascular endothelial growth factor (VEGF) in the c-Jun N-terminal kinase (JNK) and the NF-KB signaling pathways [11]. Also, the inflammatory cells of the tumor microenvironment, consisting primarily of tumor-related macrophages, can secrete TNF-α continuously to promote tumor formation, invasion, and metastasis via activation of protein-1 (AP-1) and the NF-KB pathway [12]. Our *in vitro *experiments show that UTI can inhibit the proliferation and invasion of MCF-7 human breast carcinoma cells [9] and the growth of MDA-MB-231 (present study). Taken together, these effects could be related to the down-regulation of MMP-9 in breast carcinoma cells by UTI [13]. We show here that both UTI and TAX inhibit the expression of TNF-α.

Ulinastatin (UTI) and docataxel (Taxotere, TAX) inhibit the growth of MDA-MB-231 human breast cancer cells cultured *in vitro *and xenografted into nude mice *in vivo*. The combination of both drugs is stronger than either drug alone under the conditions tested. The growth inhibition of human breast carcinoma cells and tumors could be related to the concomitant down-regulation of IL-6, IL-8, and TNF-α in breast carcinoma cells by these drugs.

## Competing interests

The authors declare that they have no competing interests.

## Authors' contributions

XZ did the MTT essay and immunohistochemistry, XS did the Cell-culturing, submitted paper and revised the paper, FG did the medical statistics, JL cultured the cell and did PCR, ZS designed this experiment and wrote this paper. All authors read and approved this final draft.
